# Predicting glaucoma progression using deep learning framework guided by generative algorithm

**DOI:** 10.1038/s41598-023-46253-2

**Published:** 2023-11-15

**Authors:** Shaista Hussain, Jacqueline Chua, Damon Wong, Justin Lo, Aiste Kadziauskiene, Rimvydas Asoklis, George Barbastathis, Leopold Schmetterer, Liu Yong

**Affiliations:** 1 Institute of High Performance Computing, A*STAR, Singapore, Singapore; 2 Singapore Eye Research Institute, Singapore National Eye Centre, Singapore, Singapore; 3 Academic Clinical Program, Duke-NUS Medical School, Singapore, Singapore; 4 SERI-NTU Advanced Ocular Engineering (STANCE) Program, Singapore, Singapore; 5 Institute of Molecular and Clinical Ophthalmology, Basel, Switzerland; 6 ETH Zurich, Zurich, Switzerland; 7 Clinic of Ears, Nose, Throat and Eye Diseases, Institute of Clinical Medicine, Faculty of Medicine, Vilnius University, Vilnius, Lithuania; 8 Department of Eye Diseases, Vilnius University Hospital Santaros Klinikos, Vilnius, Lithuania; 9 Department of Mechanical Engineering, Massachusetts Institute of Technology, Cambridge, MA USA; 10 Singapore-MIT Alliance for Research and Technology (SMART) Centre, Singapore, Singapore; 11 Department of Ophthalmology, Lee Kong Chian School of Medicine, Nanyang Technological University, Singapore, Singapore; 12 School of Chemistry, Chemical Engineering and Biotechnology, Nanyang Technological University, Singapore, Singapore; 13 Department of Clinical Pharmacology, Medical University of Vienna, Vienna, Austria; 14 Center for Medical Physics and Biomedical Engineering, Medical University of Vienna, Vienna, Austria

**Keywords:** Engineering, Diseases, Retinal diseases, Health care

## Abstract

Glaucoma is a slowly progressing optic neuropathy that may eventually lead to blindness. To help patients receive customized treatment, predicting how quickly the disease will progress is important. Structural assessment using optical coherence tomography (OCT) can be used to visualize glaucomatous optic nerve and retinal damage, while functional visual field (VF) tests can be used to measure the extent of vision loss. However, VF testing is patient-dependent and highly inconsistent, making it difficult to track glaucoma progression. In this work, we developed a multimodal deep learning model comprising a convolutional neural network (CNN) and a long short-term memory (LSTM) network, for glaucoma progression prediction. We used OCT images, VF values, demographic and clinical data of 86 glaucoma patients with five visits over 12 months. The proposed method was used to predict VF changes 12 months after the first visit by combining past multimodal inputs with synthesized future images generated using generative adversarial network (GAN). The patients were classified into two classes based on their VF mean deviation (MD) decline: slow progressors (< 3 dB) and fast progressors (> 3 dB). We showed that our generative model-based novel approach can achieve the best AUC of 0.83 for predicting the progression 6 months earlier. Further, the use of synthetic future images enabled the model to accurately predict the vision loss even earlier (9 months earlier) with an AUC of 0.81, compared to using only structural (AUC = 0.68) or only functional measures (AUC = 0.72). This study provides valuable insights into the potential of using synthetic follow-up OCT images for early detection of glaucoma progression.

## Introduction

Glaucoma is a group of progressive eye diseases, characterized by degeneration of retinal ganglion cells (RGC) and thinning of retinal nerve fiber layer (RNFL) resulting in visual field (VF) defects^1^. It is the leading cause of blindness worldwide^2^, with 111.8 million cases expected by 2040^3^. The progressive glaucomatous damage involves RGC loss in the asymptomatic stages of the disease followed by VF defects at the more advanced stage of glaucoma with significant RGC death. Although treatment is available for reducing intraocular pressure (IOP) it is difficult to customize individual treatment regimens because of the challenges in predicting the risk of progression. Hence, there is a considerable interest to use artificial intelligence (AI) to predict progression rates and facilitate immediate treatment for early-stage glaucoma patients and provide a means to identify rapidly progressing patients who are at high risk of visual disability and may therefore require escalation in treatment.

Progression can be assessed based on functional and/or structural parameters. Structural parameters associated with the RNFL and ganglion cell-inner plexiform layer (GCIPL) complex^4,5^ can be obtained using imaging of the optic nerve head (ONH), macula, and surrounding regions using optical coherence tomography (OCT)^6^. The most commonly used functional test of glaucoma is based on standard automated perimetry, which is regarded as the clinical gold standard for assessing visual function. Clinicians use this test to assess the regions of a patient’s field of vision affected by glaucoma and the severity of vision loss^4^. However, this assessment is challenging due to VF variability which can be due to several factors like cataracts, the severity of glaucoma with frequent fixation losses, learning effects or distraction^7^. Hence, several studies have combined structural and functional data to address the VF variability issue and improve glaucoma detection performance as well as functional progression^8,9^.

Machine and deep learning models have been used for the classification of glaucoma based on fundus images^10–12^ as well as OCT^13^. The majority of OCT-based models used parameters extracted from segmented images as input for the AI-based prediction of VFs. This limits the generalizability of deep learning models to pre-defined structural features which are prone to errors because of segmentation issues. The ability of these models to discover new structural biomarkers, which are not quantified by the scanners, is also limited. Recently, several authors have developed techniques using convolutional neural networks (CNNs) to directly input 2-D or 3-D information from OCT images to predict VF^14–17^.

AI has also been utilized for predicting glaucoma progression. Different unsupervised and supervised machine learning models including random forests, Bayesian techniques, and recurrent neural networks (RNN) have been used to model glaucoma progression^18–23^. A deep learning model was used to estimate longitudinal changes in RNFL thickness from fundus photographs in order to predict the future development of glaucomatous visual field defects^24^. Yousefi et al.^25^ combined structural data with visual field inputs and reported that the accuracy of machine learning classifiers in discriminating stable versus progressing glaucoma patients did not improve when VFs were complemented with RNFL data. However, Garway-Heath et al. showed that glaucoma progression rates could be estimated with higher accuracy by combining VF and OCT data compared to only VF data^8^. Similar results were obtained by Dixit et al. by using a convolutional long short-term memory (LSTM) model for identifying glaucoma progression and showing that supplementing VF data with basic clinical data (cup-to-disc ratio, corneal thickness, and IOP) could improve the performance of the predictive model^9^.

Some studies on glaucoma as well as other diseases have attempted to model expected disease progression on patient images directly, through the use of generative models like generative adversarial networks (GAN) and variational autoencoders (VAE) in estimating disease-relevant images at certain future time points in order to predict the disease progression. This was achieved by leveraging on the ability of GAN to translate images from the source to the target domain with high precision and has been applied to MRI images^26,27^, radiographs^28^ and OCT images^29^. In glaucoma, one study used conditional GAN architecture to predict glaucoma progression by reconstructing cross-sectional OCT images from three or two prior measurements^30,31^. A GAN-based approach was used to learn to translate fundus images to corresponding OCT images, after which the generated images were used for early glaucoma detection^32^. Another glaucoma study used VAE for modelling spatiotemporal data corresponding to longitudinal visual fields from a cohort of glaucoma patients^33^.

In this paper, we propose a glaucoma progression prediction framework consisting of multimodal deep-learning model aided by a generative algorithm. The proposed method is used to assess if synthesized follow-up OCT images can boost the accuracy of predicting glaucoma progression.

## Results

### Multimodal dataset

**Figure 1:**
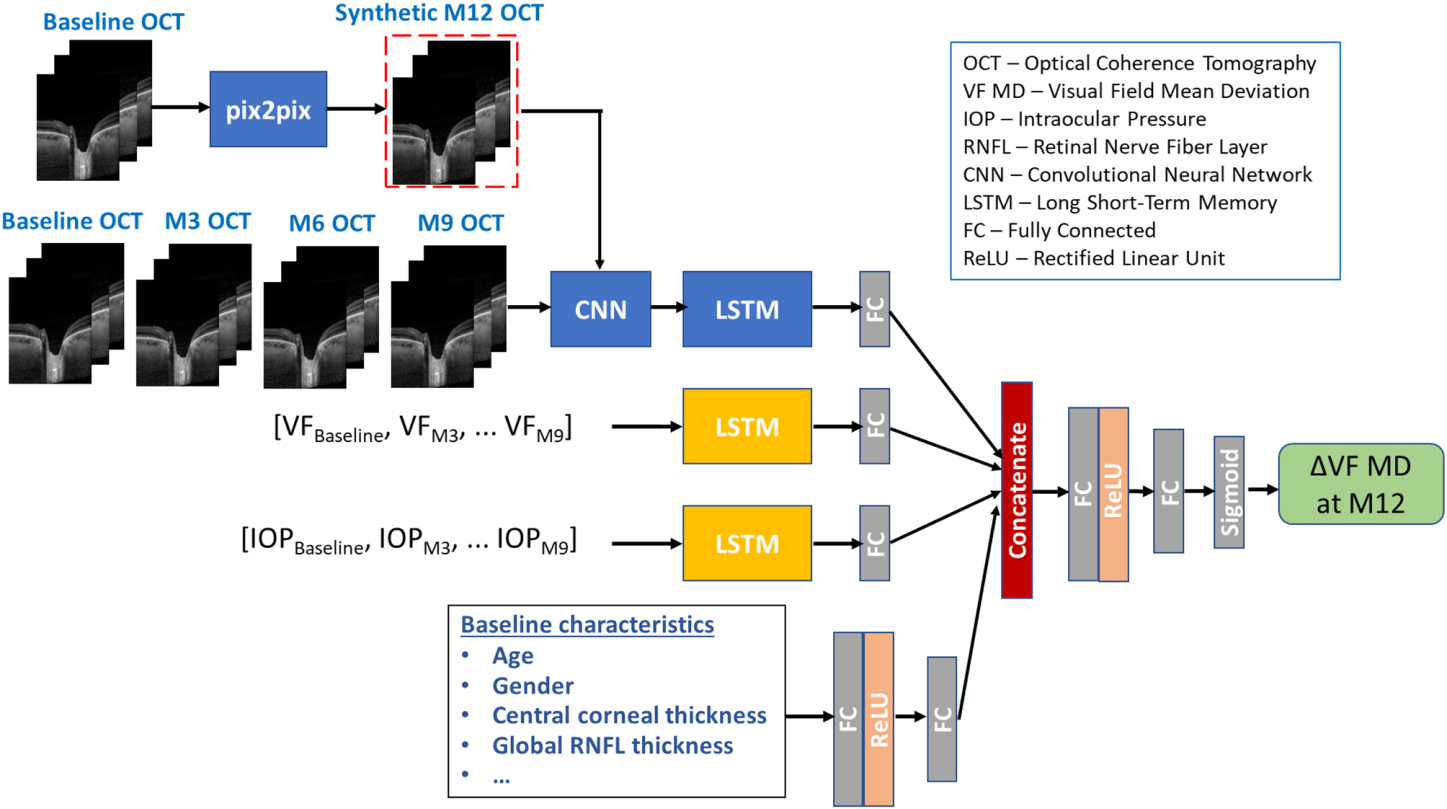
Progression prediction framework using OCT images, VF MD values, IOP and patient baseline characteristics to predict the slow vs fast progression of glaucoma patients 12 months (M12) after the baseline visit. The framework comprises CNN (ResNet-34) for feature extraction from OCT images, LSTM models to learn the temporal relationships within longitudinal inputs and pix2pix GAN for generating the M12 images using baseline images.

The longitudinal dataset used for training comprised measurements from 105 glaucomatous eyes. This number reduced to 86 after removing the cases with incomplete patient visit data. This longitudinal multimodal patient dataset comprised baseline patient characteristics and OCT images, VF MD and IOP values measured at five visits—baseline, and at 3 months (M3), 6 months (M6), 9 months (M9) and 12 months (M12). The demographic and clinical characteristics of the patients at baseline are presented in Table 1. A deep learning-based prediction pipeline, aided by a generative model (Fig. 1) was used to predict the glaucoma progression. Our approach involved predicting change in VF MD (∆VF) at M12 with respect to baseline by utilizing the longitudinal multimodal patient data. For this, we formulated a binary classification problem, where ∆VF values were divided into 2 classes, with ∆VF > − 3 dB categorized as slow glaucoma progressors (Class-1) and ∆VF < − 3 dB as fast progressors (Class-2). Specifically, we have used baseline, M3, M6 and M9 multimodal data, combined with synthetic future OCT images (from pix2pix GAN) to predict ∆VF at M12.

**Table 1:** Demographic and clinical characteristics pf patients at baseline.

Baseline characteristic	Range
Age, years	67.28 ± 8.92
Gender (female:male)	47:53
BCVA, decimal scale	0.68 ± 0.25
REFR, D	− 0.40 ± 1.85
CCT, µm	519*.*45 ± 31*.*22
AXL, mm	23.60 ± 0.93
IOP, mmHg	27.36 ± 6.69
VF MD, dB	− 14.68 ± 8.43
Global RNFL thickness, μm	53.38 ± 13.62

*BCVA* best-corrected visual acuity, *REFR* refractive error, *D* diopters, *CCT* central corneal thickness, *AXL* axial eye length, *IOP* intraocular pressure, *VF* visual field, *MD* mean deviation, *dB* decibels, *RNFL* retinal nerve fiber layer. Values expressed as mean ± standard deviation, unless otherwise indicated.

**Figure 2:**
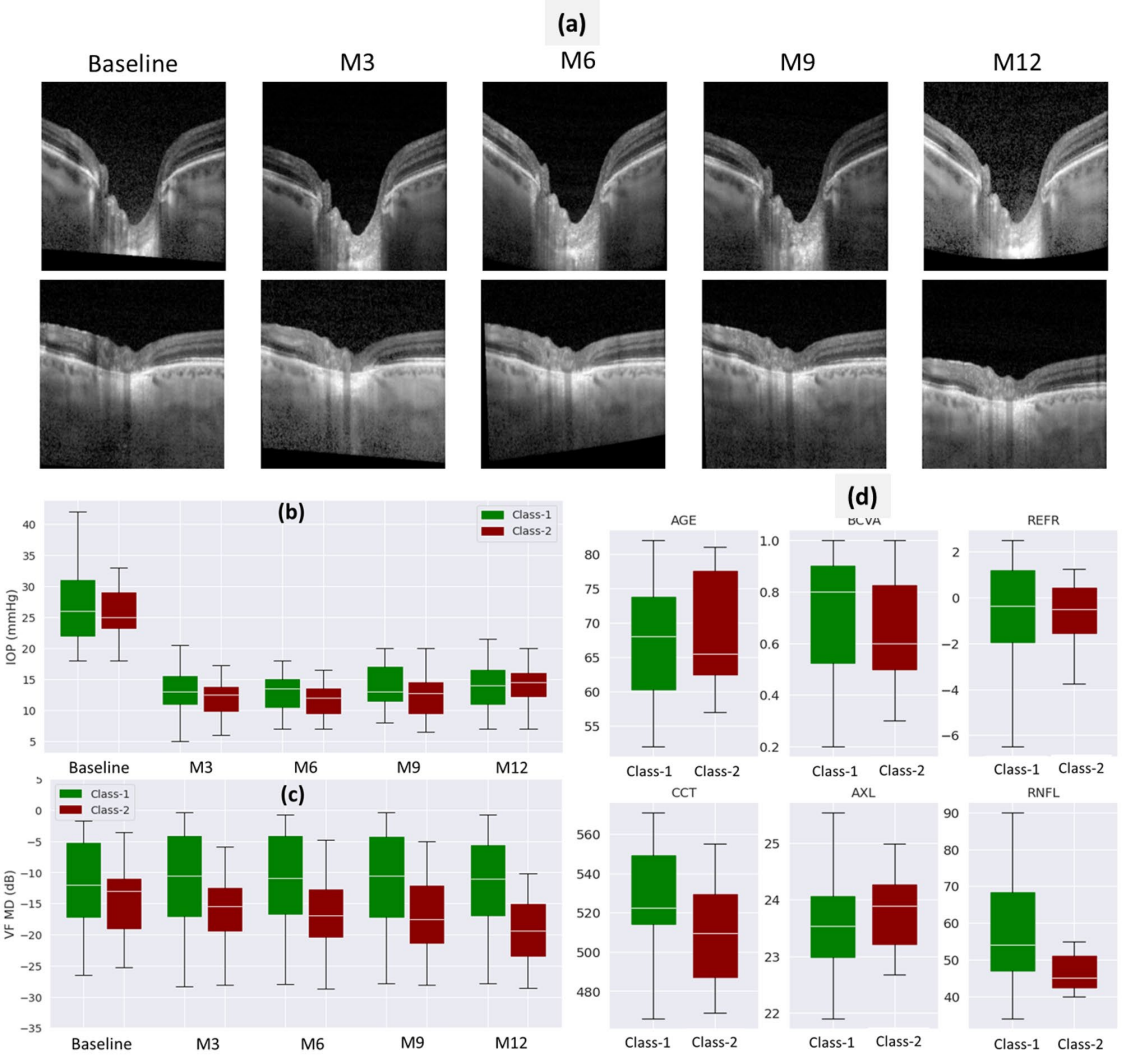
Multimodal longitudinal (**a**–**c**) and baseline inputs (**d**) used for training the progression prediction model. (**a**) Examples of OCT images used for glaucoma progression prediction task. Each row corresponds to OCT B-scans of a patient at the five visit times (Baseline and M3-M12) over 12 months. (**b**) The IOP distributions at baseline and M3–M12 visit times for two glaucoma classes used in this work, where Class-1 (green) refers to slow progressing cases (∆VF > − 3 dB) and Class-2 (red) refers to fast progressing cases (∆VF < − 3 dB). (**c**) VF MD distributions for Class-1 and Class-2 patients at the five visit times. (**d**) Distributions of baseline demographic and clinical features—age (years), best-corrected visual acuity (BCVA in decimal scale), refractive error (REFR in D), central corneal thickness (CCT in µm), axial eye length (AXL in mm), retinal nerve fiber layer (RNFL in µm) thickness of patients belonging to Class-1 and Class-2 progressing classes.

Figure 2a shows examples of OCT B-scans of two patients (rows) at five visit times, i.e. baseline, M3, M6, M9 and M12 (columns). These images were used for training the progression prediction model by first extracting OCT image features using a pre-trained ResNet-34 architecture backbone. This step resulted in a 512-dimensional feature vector for all OCT B-scan patient images across different visits.

Figure 2b–d show the distributions of demographic and clinical features listed in Table 1. The IOP distributions in Fig. 2b show the IOP lowering associated with trabeculectomy performed after the baseline visit. We can also see that the median IOP values of Class-1 patients are higher than that of Class-2 patients at all visits except at M12. Figure 2c shows that there is improvement in VF MD values after the baseline visit for Class-1 patients, while Class-2 patients experience a decline in VF. Moreover, the fast progressing Class-2 patients have lower initial VF values than the Class-1 patients, indicating that more advanced cases of glaucoma decline faster. Figure 2d shows the distributions of some baseline clinical features of patients in the two glaucoma progressor classes. In terms of the differences between the two classes, fast progressing Class-2 patients have lower best-corrected visual acuity (BCVA), lower central corneal thickness (CCT), higher axial eye length (AXL), lower RNFL thickness as compared to Class-1 patients.

### Model training and testing strategy

For training the deep learning pipeline, the data samples were split into 75% for training and 25% for testing at the patient level, i.e. 65 patient samples in the training dataset and 21 in the test dataset. The training samples were further used for 5-fold cross-validation based training. The choice of − 3 dB threshold for classifying patients into slow and fast progressing cases led to the patient dataset consisting of fewer cases of fast progressing patients (12/86), with 56 Class-1 and 9 Class-2 patients in the training set and 18 Class-1 and 3 Class-2 patients in the test set. This problem of class imbalance was addressed by adopting the following strategies:

(i) Oversampling by randomly duplicating samples from the under-represented minority class (Class-2) during training. This resulted in 44 Class-2 samples in the training set and 12 in the validation set.
(ii) Focal loss instead of binary cross-entropy loss during model training. In case of class imbalance, the cross-entropy loss function gets overwhelmed by the majority class “easy” samples, leading to the model performing well for majority class and poorly on the “hard” minority samples. The use of focal loss addresses class imbalance by down-weighting the easy samples, such that the model can focus on learning the hard samples.
(iii) Performance metrics which are more meaningful for a class imbalance problem. We used confusion matrix, F1 score and area under the ROC curve (AUC) instead of classification accuracy.

Moreover, due to the small size of the dataset, we trained our model on OCT B-scans instead of OCT volumes, where each volume consisted of 49 slices. This resulted in a total of 4214 OCT B-scans for model training and testing. The model prediction was obtained at the B-scan slice-level, which was then converted to patient-level outcome.

As mentioned earlier, focal loss was used for the progression classification task. Focal loss adds a modulating term to the standard cross entropy loss in order to reduce the impact of easy samples on the loss function and focus on hard samples from the minority class. The focal loss is defined as:

1 $$FL{(p}_{t})=-{\left(1-{p}_{t}\right)}^{\gamma }{\mathrm{log}}({p}_{t})$$

where ${(1-{p}_{t})}^{\gamma }$ is the modulating term, $\gamma$ is the focusing parameter and ${p}_{t}$ is the model’s estimated probability for class with label y = 1 (Class-2 in our case). By setting $\gamma$ > 0, the loss for easy, well-classified samples (${p}_{t}>0.5$) is down-weighted, while the loss for hard, misclassified examples with small ${p}_{t}$ remains unaffected. We used $\gamma =2$ for this work.

The Adam optimizer was used with a learning rate of 0.001 which was linearly reduced by a factor of 0.9 if there was no reduction in the validation loss for 5 epochs. The training and validation batch size was 32 and the model was trained for 10 epochs. After training, the performance of the model was evaluated on the test dataset by computing metrics like Area Under the Receiver Operating Characteristic (ROC) curve (AUC), confusion matrix and F1 score. AUC is used to measure the performance of a model for classification problems, in terms of the degree of separability of different classes. The ROC curve is plotted as true positive rate (TPR) against false positive rate (FPR), where:

2 $$TPR=\frac{TP}{TP+FN}$$

3 $$FPR=\frac{FP}{TN+FP}$$

A confusion matrix can be used to evaluate the performance of an ML classification model by comparing the actual labels (Class-1 and Class-2 in our case) with those predicted by the model. F1 score is used to assess the class-wise predictive performance of a model and is defined as the harmonic mean of precision and recall where precision refers to the percentage of correctly predicted positive samples out of all the samples predicted as positive, and recall is a measure of how many positive samples are correctly predicted out of all the actual positive samples.

All the experiments in this paper were conducted on an Ubuntu 20.04 server with two GeForce RTX 3090 GPUs with Cuda 10.2 platform, 10-core Intel Xeon CPU (W-2255 3.70 GHz) and 128 GB memory. We used Python 3.7.10 distributed with Anaconda 4.13.0 (64-bit) to implement deep learning models using the PyTorch library.

### Importance of input modalities

**Figure 3:**
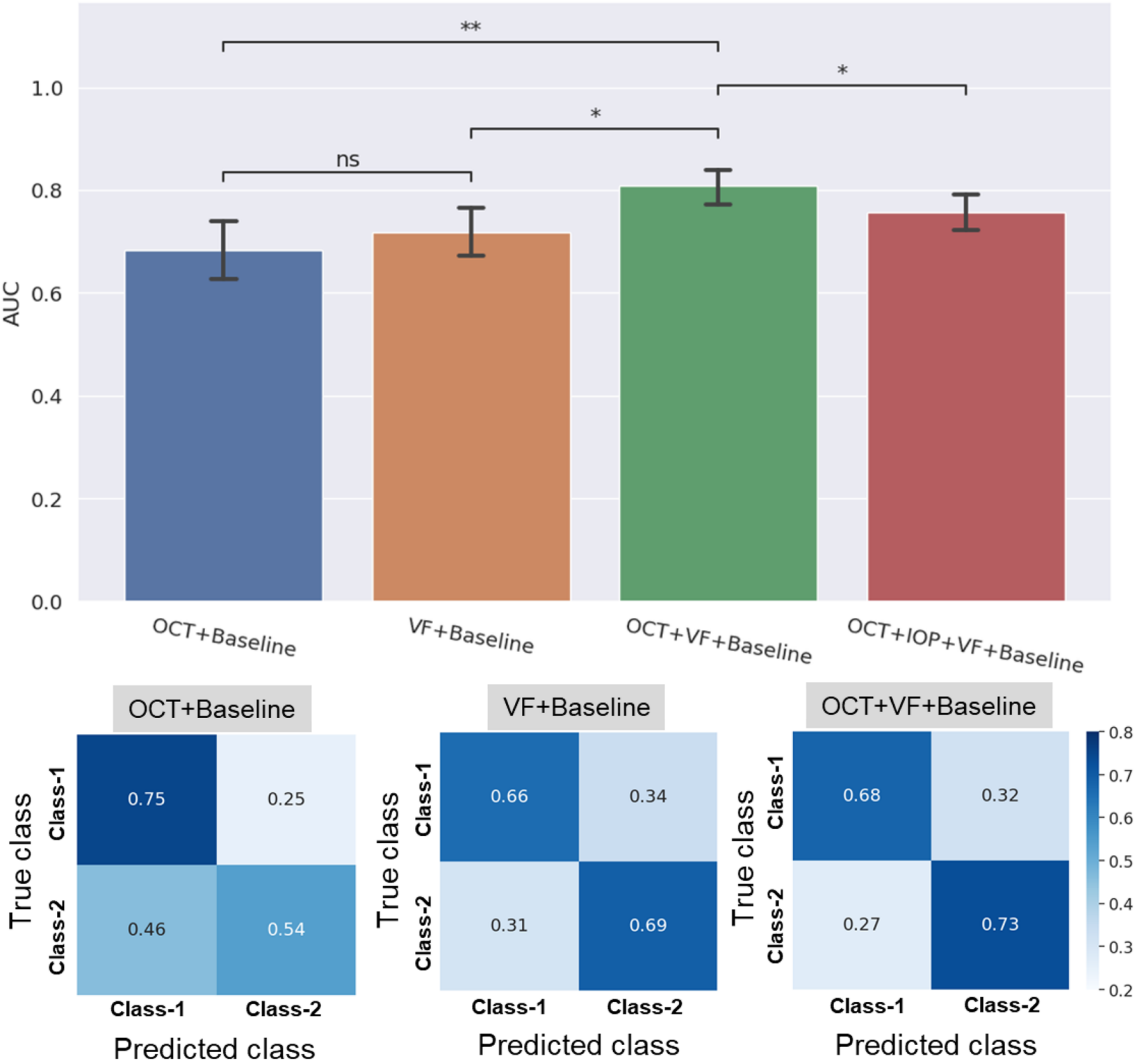
Top panel shows the prediction AUCs obtained when baseline demographic and clinical data is used with only OCT images (blue), only VF MD values (orange), combined OCT and VF MD inputs (green), and OCT images combined with VF MD and longitudinal IOP values (red). Statistical annotations are as follows: **P value < 0.01, *P value < 0.05 and ns denotes “not statistically significant”. Confusion matrices shown in the bottom panel for three input combinations to the progression model suggest that the “OCT + VF + Baseline” input combination performs well for both Class-1 and Class-2 patients, correctly predicting 73% of the fast progressing cases (Class-2).

Firstly, we compared the effects of different modalities on visual loss prediction performance. For this, we combined the baseline demographic and clinical patient data (Table 1) with the time-series inputs of OCT image features, VF MD and IOP values until M9 to predict ∆VF at M12. We compared three scenarios where baseline patient data was combined with (1) OCT time series image inputs, (2) VF MD time series inputs, and (3) both OCT and VF time series inputs. The top panel of Fig. 3 shows AUC scores for the classification of ∆VF into slow and fast progressing classes based on different combinations of inputs along with statistical annotations. The mean AUC corresponding to “OCT + Baseline” inputs is 0.68 and F1 score is 0.73. “VF + Baseline” leads to increase in AUC and reduction in F1 score, but these changes are not statistically significant. However, when longitudinal OCT images and VF values are combined with the baseline inputs (“OCT + VF + Baseline”), the prediction AUC increases significantly to 0.81, with a P value of 0.002 compared to the AUC obtained with “OCT + Baseline” inputs, and P value = 0.014 when compared to “VF + Baseline” inputs. Further, when longitudinal IOP values are also combined with OCT, VF and baseline data, the mean AUC drops significantly to 0.76, and increase in F1 score to 0.74 is not significant.

Figure 3 also shows the confusion matrices for the three cases of “OCT + Baseline”, “VF + Baseline” and “OCT + VF + Baseline”. As shown, “OCT + Baseline” input combination performs best for the slow progressing Class-1 patients while it doesn’t do well for the fast progressing Class-2 patients, failing to correctly predict almost 50% of the patients. “VF + Baseline” inputs yield similar performance for both Class-1 and Class-2 samples. When OCT and VF inputs are combined (“OCT + VF + Baseline”), the model performs well for Class-1 samples and gives best performance for Class-2 patients, correctly predicting 70% of the cases while also misclassifying 30% of Class-1 patients as belonging to Class-2 (false positive error). Hence, this combination of inputs can predict the vision loss for slow progressing patients and can also achieve the best prediction for more critical fast progressing patients. Based on these results, we found that “OCT + VF + Baseline” achieves the best AUC for predicting slow vs fast vision loss as well as the best prediction performance for fast vision loss. Hence, we chose it as the input combination for further analysis in the rest of the paper.

### Early prediction of visual loss

**Figure 4:**
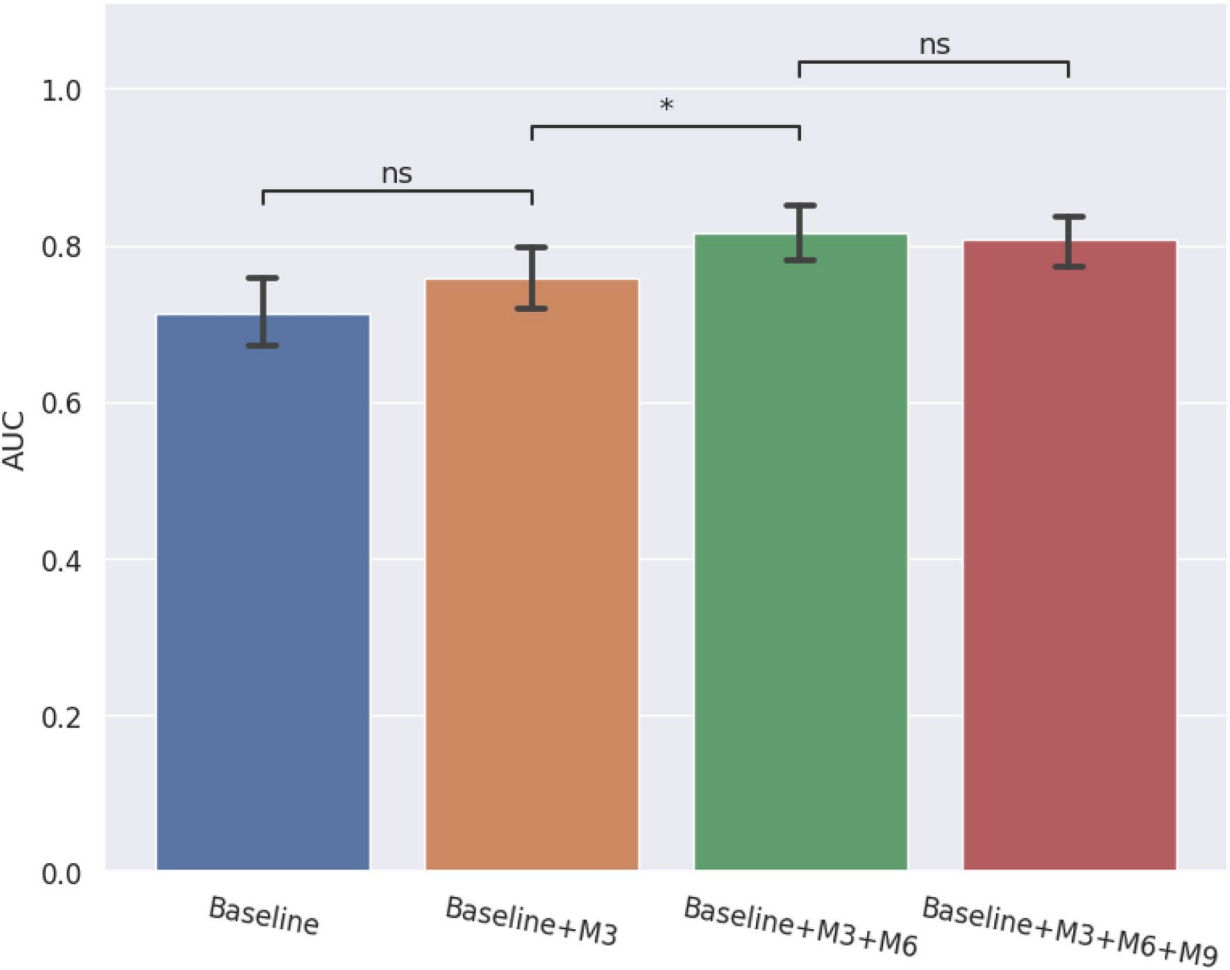
AUC for progression prediction by utilizing multimodal inputs comprising baseline patient inputs, OCT images and VF MD values at different time-points of patient visits from baseline (blue) until M3 (orange), M6 (green) and M9 (red). Statistical annotations are as follows: *P value < 0.05 and ns denotes “not statistically significant”.

Next, we performed progression prediction by utilizing the multimodal inputs at different time-points of patient visits. To determine how early our glaucoma progression model can predict the visual loss, we trained the model based on baseline patient characteristics combined with OCT images and VF MD values from baseline until M3, M6 and M9. As shown in Fig. 4, when only baseline visit information is available, AUC = 0.71, which increases to 0.76 when M3 visit information is also included. The AUC further increases significantly to 0.82 (P value = 0.013) when M6 inputs are combined with the previous visit inputs. As more near future information is included, AUC doesn’t improve further, with AUC = 0.81 (P value = 0.662) achieved by including M9 visit inputs. This can be explained by noting that as we add data at more time points closer to the future, the progression model has extracted enough information at some point of time (M6 in our case) to achieve significantly accurate prediction of vision loss and more temporal data (M9 data) doesn’t further improve the model performance significantly.

Hence, our progression prediction model can perform visual loss prediction 6 months ahead in time with AUC = 0.82 and F1 score = 0.76 by classifying patients into slow and fast glaucoma progressors.

### Progression prediction based on synthetic future OCT images

**Figure 5:**
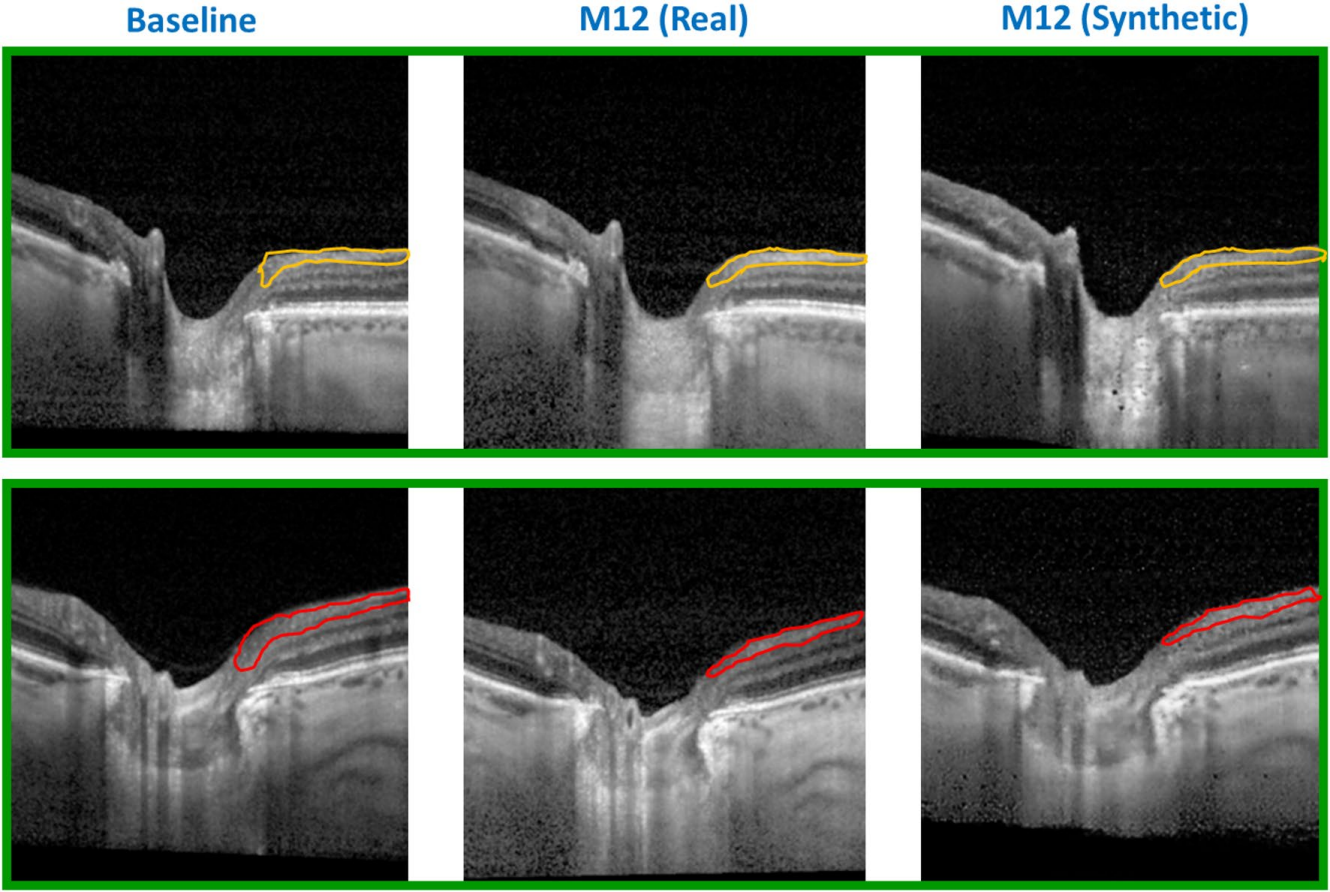
Real and pix2pix GAN based synthesized OCT B-scans of two patients (rows) showing baseline (left), real M12 (center) and synthetic M12 (right) B-scans. The RNFL thinning in a slow progressing case (top row) and a fast progressing case (bottom row) is demarcated by orange and red coloured outlines respectively. The thinning of RNFL as shown from the baseline to M12 images is also captured well by the synthetic M12 images.

We used pix2pix GAN to: (a) synthesize OCT B-scans at a future time point, and (b) to utilize the synthetic future B-scans in the progression prediction pipeline and investigate if it helps to improve the prediction accuracy of ΔVF at M12. Specifically, we generated M6, M9 and M12 OCT images conditioned on the baseline images. We evaluated the use of synthetic OCT images for accurate and early glaucoma prediction by considering both time of prediction and the corresponding AUC. Figure 5 shows real and synthesized OCT B-scans of two patients (rows). The baseline (left), real M12 (center) and synthetic M12 (right) B-scans are shown highlighting the thinning of RNFL (coloured demarcations) between baseline and real M12 images, which have been captured by the synthetic M12 images. In the top row, the baseline and M12 images of a slow progressing glaucoma patient show the RNFL thinning with an orange boundary, which is replicated well in the synthetic M12 image. Similarly, the red boundaries in the bottom row point to the thinning of RNFL in a fast progressing patient, shown as a change occurring from baseline to M12, as captured by both real and synthetic M12 images.

**Figure 6:**
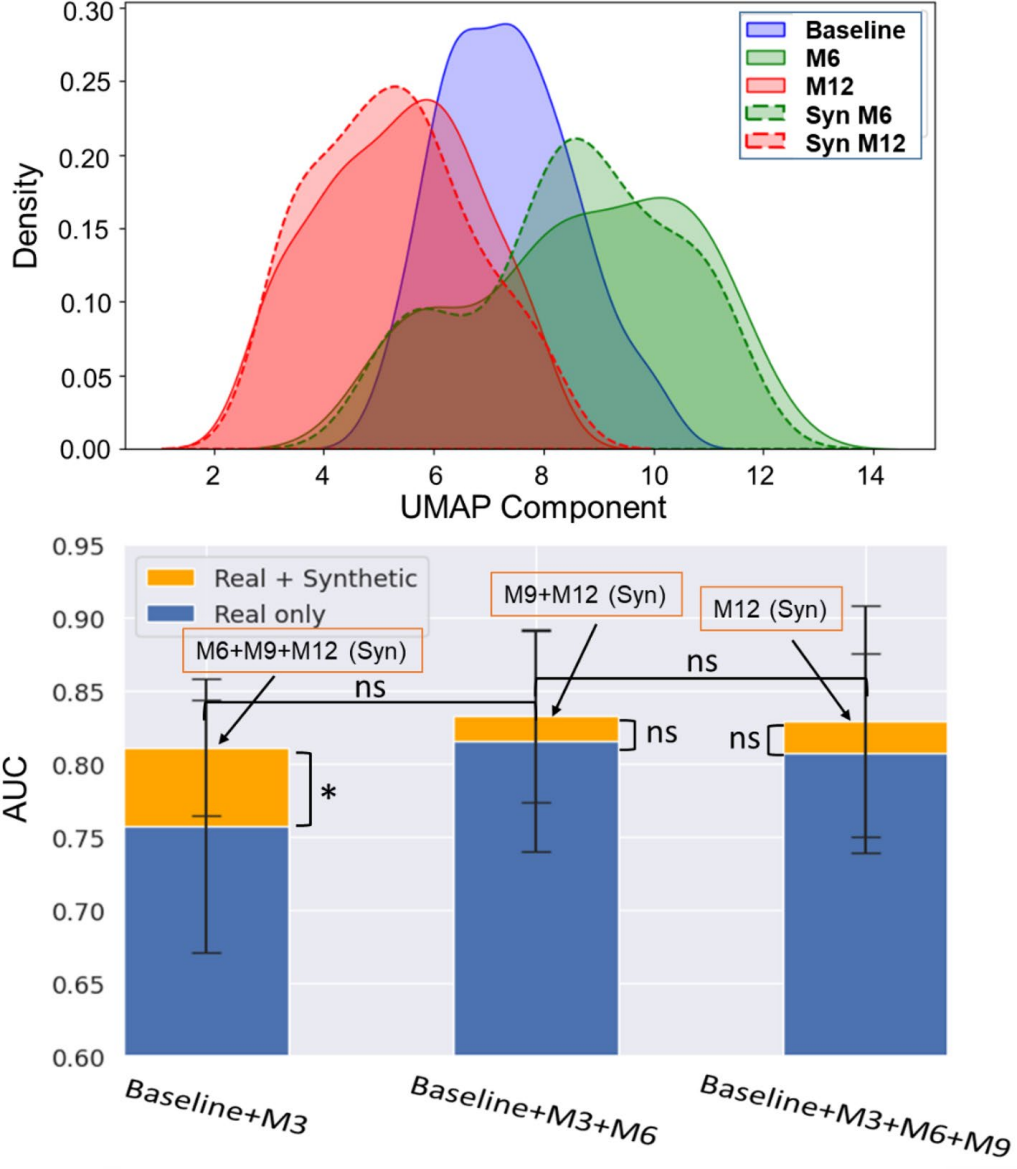
Probability density distribution for OCT image features (top panel) corresponding to baseline (blue), real and synthetic M6 images (green) and real and synthetic M12 images (red). AUCs obtained without (blue bars) and with (orange bars) synthetic OCT images (bottom panel). * indicates P value < 0.05 and ns denotes “not statistically significant”.

Next, we looked at the feature distributions of real and synthetic OCT images extracted using pre-trained ResNet-34. We used Uniform Manifold Approximation and Projection (UMAP) method to learn low dimensional representation of the image features and then visualized the first dimension of the transformed features. Figure 6 (top) shows the feature distributions of real baseline, M6 and M12 OCT images (solid boundary) plotted alongside the distributions of synthetic M6 and M12 images (dashed boundary). The real and synthetic image feature distributions corresponding to both M6 (green) and M12 (red) show significant overlaps. We see that there is a greater overlap between real and synthetic image distributions at the same time point (M6/M12) than between the baseline (blue) and M6/M12 distributions. To compute the similarity/distance between these feature distributions, we performed the Kolmogorov–Smirnov (KS) test and obtained P value to determine if the two samples are significantly different. P value for KS test of both baseline vs real/synthetic M6 and baseline vs real/synthetic M12 features is < 0.001, while for real vs synthetic M6 images, P value = 0.557, and for real vs synthetic M12 images, P value = 0.678. Hence, we can conclude that the baseline image distribution is significantly different from both real and synthetic M6/M12 image distributions, while real and synthetic M6/M12 images have similar feature distributions suggesting that the generated images are realistic.

Next, we employed the synthetic OCT images to predict glaucoma progression. To do this, we trained the model on real images and tested it on synthetic images in the following manner. We started from our best results in the previous section, which showed that ∆VF prediction can be done at M6 with the best AUC of 0.82. Further, we investigated if we could improve this result and/or achieve similar performance at an earlier time point with the help of synthetic future OCT images. We took the multimodal visit data from baseline until M6 and combined it with real M9 and M12 OCT images for training the progression model. The trained model was then tested using synthetic M9 and M12 images combined with prior data. We repeated this experiment for previous time point, M3, to test for early prediction and next time point, M9, to test for improved performance. As shown in Fig. 6 (bottom), when M9 and M12 synthetic images are combined with visit data until M6, the AUC increases from 0.82 (blue bars, same as Fig. 4) to 0.83 (orange bars, real + synthetic data). However, this improvement is statistically not significant with P value of 0.478. When ΔVF prediction is done at the previous time point M3, AUC increases significantly from 0.76 (only real data) to 0.81 (real + synthetic M6, M9, M12) with a P value of 0.038. Similar to the trend seen at M6, when prediction is done at M9, synthetic images fail to contribute to any significant improvement in AUC compared to when only real images are used.

**Figure 7:**
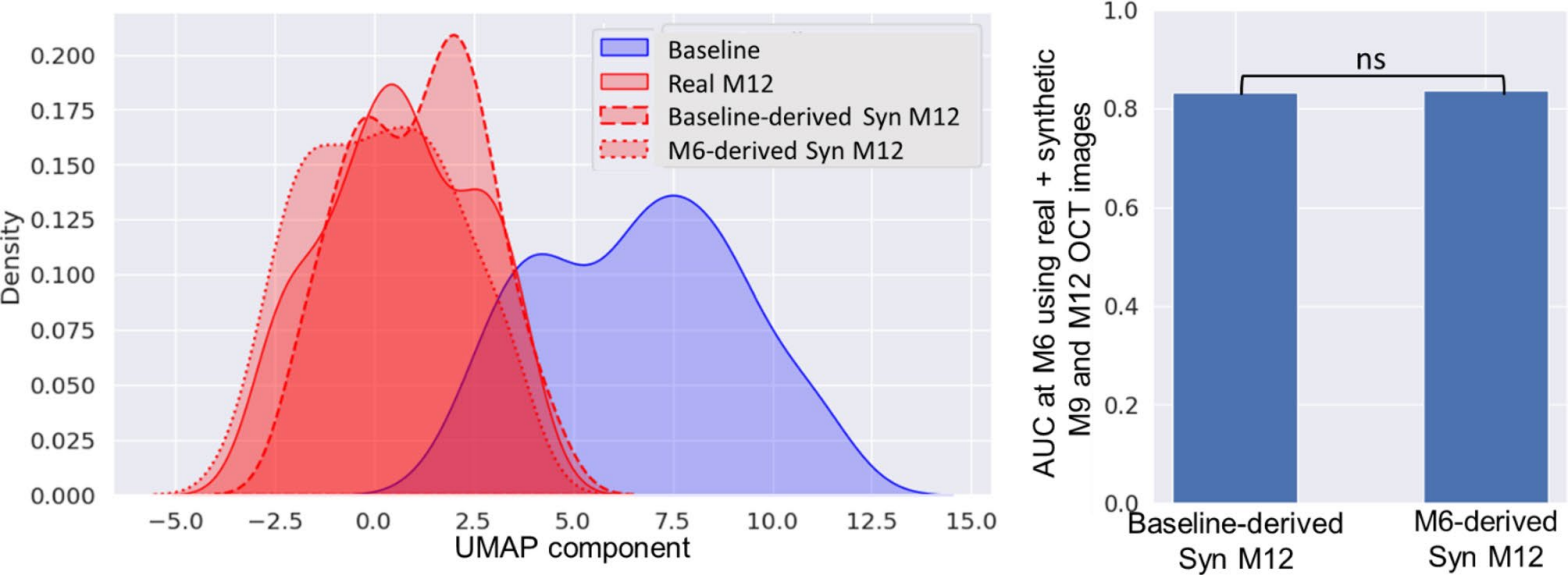
Real and synthetic OCT image feature distributions (left) showing that M12 images synthesized using baseline images (dashed red) and M6 images (dotted red) have similar distributions, which are significantly different from baseline image distribution (blue). The AUCs obtained using synthetic images derived from baseline and M6 images are similar (right). ns denotes “not statistically significant”.

Since, the progression model achieved the best AUC (= 0.83) at M6 with the help of synthetic M9 and M12 images, we also investigated if M6 instead of baseline images can be used to synthesize more realistic future images. To test this, we conducted an experiment where future M9, M12 OCT images were synthesized using the real M6 images. To compare the synthetic images generated using baseline and M6 images, we have plotted the image feature distributions (low dimensional representation using UMAP), which show that the synthetic M12 images generated using both baseline (dashed red) and M6 (dotted red) images have similar feature distributions as that of real M12 images (solid red), with P values of 0.066 and 0.337 respectively (Fig. 7, left). These real and synthetic M12 distributions are significantly different from the baseline image distribution (blue, P value < 0.001). Further, we also used the synthetic M12 images conditioned on M6 real images to predict the vision loss at M12. The AUC obtained when the progression model used real data until M6 combined with M6-derived synthetic M9 and M12 OCT images to predict the class of ΔVF, was 0.84, which is very similar (P value = 0.934) to the AUC (= 0.83) when baseline images were used to generate M9 and M12 synthetic images, as shown in Fig. 7 (right).

The high similarity between synthetic images generated using real baseline and real M6 images is due to very small changes in the OCT images of patients from one visit to another. As seen in Fig. 2a, the longitudinal OCT B-scans of patients show very little change across visits, which can be attributed to the fact that these patients are advanced glaucoma patients and may have already experienced significant structural changes in their retina, as captured by the OCT imaging method.

Based on these results, we can conclude that our progression model aided by synthetic images can predict ΔVF at M3, i.e. 9 months ahead in time, with a statistically significant mean AUC of 0.81 compared to progression model without synthetic images, which achieves a mean AUC of 0.82 (P value = 0.850) later at M6, i.e. 6 months ahead in time. Hence, the use of synthesized future OCT images can enable early and accurate glaucoma prediction.

## Discussion

Our study aimed to develop a framework for predicting glaucoma progression by generating future OCT images and predicting visual loss. The key contributions of our work are: (1) the first glaucoma prediction model using multimodal data, including OCT images, VF values, and baseline demographic and clinical data; (2) the first use of synthetic OCT images in a progression prediction pipeline for enhancing prediction accuracy. Specifically, we used a pix2pix GAN to synthesize OCT images at 6 months (M6), 9 months (M9) and 12 months (M12) after the first patient visit, and then employed a CNN-LSTM network to predict changes in VF MD at the M12 visit based on patient data from earlier time-points combined with the synthesized images. Our approach is highly effective in accurately predicting fast or slow progressors, with the best AUC of 0.83. This is a significant improvement over existing studies that use only structural or functional measures and have lower AUC values. The findings of this study suggest that our approach has the potential to improve the early detection of glaucoma progression, leading to better patient outcomes and potentially reducing the risk of vision loss.

Most of the earlier work on glaucoma progression prediction employed traditional machine learning models including random forest, support vector machine and naïve Bayes classifier^20^. The majority of these studies were based on visual functional inputs like SAP-measured VF sensitivity at various locations and global parameters like MD and pattern standard deviation (PSD)^18,20,21^. Very few studies have used only structural inputs like OCT measurements combined with demographic/clinical data to detect visual field progression in glaucoma patients^22^. In contrast, traditional machine learning models like random forest and Bayesian modeling approaches have been used to combine structural and functional measures in order to improve glaucoma prediction^23–25^. Deep learning applications in predicting glaucoma progression have used CNN and RNN based on VF and clinical data^9,34,35^. While existing AI-based glaucoma prediction models utilize a combination of structural and functional inputs, none of these methods use OCT images directly as input to the models. Here, we show how our progression prediction model compares with some of these earlier works. Since, we could not access the datasets used in these studies, we applied their methods on our glaucoma dataset to predict the VF loss 12 months after the first patient visit (Table 2). We also present the AUCs and 95% confidence intervals (CI) obtained by our model using different combinations of inputs and the corresponding P values.

**Table 2:** Glaucoma progression prediction AUCs using earlier AI-based methods as compared with our proposed method.

Modeling approach	Inputs used	AUC (95% CI)	P value
Traditional machine learning models	VF^18,20^	0.71	–
	OCT + Baseline^22^	0.61	–
	VF + OCT^25^	0.58	–
Deep learning models	VF + Baseline^9^	0.72	–
Single modal deep learning	OCT + Baseline [Current work]	0.68 (0.62, 0.74)	< 0.001
Single modal deep learning	VF + Baseline [Current work]	0.72 (0.66, 0.77)	0.003
Multimodal deep learning	OCT + VF + Baseline at M3 [Current work]	0.76 (0.71, 0.80)	0.038
Multimodal deep learning with generative model	OCT + VF + Baseline at M3 + Future Synthetic OCT [Current work]	0.81 (0.79, 0.84)	Reference
Multimodal deep learning with generative model	OCT + VF + Baseline at M6 + Future Synthetic OCT [Current work]	0.83 (0.80, 0.86)	0.264

Here “Baseline” inputs refer to the demographic and clinical parameters at the first visit as listed in Table 1, i.e. AGE, GENDER, BCVA, REFR, CCT, AXL, RNFL, IOP and VF MD. AUCs and 95% CI are listed for results achieved by our model using different input combinations, where the statistical significance was determined by performing *t*-test and computing the P values. We considered a P value of less than 0.05 as statistically significant.

Some earlier studies have utilized generative models like GAN and variational autoencoders (VAE) to enable glaucoma detection and prediction. In one study, conditional GAN was used to reconstruct cross-sectional OCT images from past patient visits for predicting glaucoma progression^26^. GAN was also used to generate corresponding OCT images from fundus images to achieve early glaucoma detection based on the generated OCT images^32^. Kumar et al.^27^ used progressively growing GAN model to generate circumpapillary OCT scans, which were then evaluated on glaucoma detection task. Berchuch et al.^33^ demonstrated the use of VAE for modeling spatiotemporal data corresponding to longitudinal visual fields from a cohort of glaucoma patients. In this work, we have explored the use of pix2pix GAN to generate future OCT images based on OCT images from the first patient visit. These synthesized future images, representative of glaucoma induced degradation, when combined with past inputs, were found to enhance the accuracy of glaucoma progression prediction model.

Our study is important because it uses a combination of structural, functional, demographic, and clinical factors to predict progression. This approach can be more clinically relevant as it represents a more holistic integration of the acquired information since there is no consensus on specific tests and measurements needed to predict glaucoma progression. The use of combined inputs enables automatic extraction of information relevant to glaucoma progression. We have developed a deep learning architecture that can incorporate multiple data sources with different modality inputs, including images, temporal and cross-sectional numeric data. The use of OCT images without relying on pre-defined structural features helps to avoid the time-consuming and error-prone image segmentation process.

A limitation of our study is the small dataset used for training and evaluating the deep learning models, and the class imbalance arising due to much smaller number of fast vs slow progressing glaucoma cases in our dataset. These factors can affect the ability of our model to identify all input features relevant for predicting glaucoma progression. Nevertheless, we used cross-validation and stratified subsets of data to ensure that both slow and fast progressing cases were represented in training and testing, and to prevent overfitting of the model on training data. Moreover, the dataset belongs to patients of a particular ethnicity, where all of them have undergone the trabeculectomy procedure for surgical lowering of IOP. This may limit the generalizability of our model and the conclusions obtained through our analysis.

In future, we will address the limitations of our study by training and testing the proposed approach on larger balanced datasets of patients belonging to early, moderate, and severe glaucoma stages. We will also apply our model on datasets from other patient cohorts to validate its robustness and generalizability. Further, we will also investigate the impact of other factors, like genetic inputs, on prediction of VF loss. Currently, our method utilizes a generative model to synthesize future OCT images, which are then used for progression prediction. We will extend the generative model to synthesize OCT images at any given time-point. This method can be used to address the problem of missing longitudinal data, which is a big challenge faced in disease progression modeling problems. Moreover, the predicted follow-up images can also aid clinicians in forecasting potential glaucoma induced changes and making more intuitive clinical decisions.

In conclusion, we developed a deep learning model using GANs for predicting VF loss in glaucoma patients based on OCT images, VF values, demographic and clinical data. The results showed that the combination of structural and functional inputs with baseline patient characteristics resulted in the highest predictive performance (AUC = 0.83) 6 months ahead in time. The use of synthetic OCT images generated using GAN also allowed us to achieve similar performance (AUC = 0.81) even earlier, at 9 months prior to the vision loss. The model's predictive ability improved with more time-series data, and it could predict visual loss early in the disease course, outperforming other existing models (AUC = 0.61–0.73). Overall, this research contributes to the growing field of AI-assisted prediction of glaucoma progression and has the potential to improve patient outcomes and reduce the burden of glaucoma on society.

## Methods

### Dataset

This work is based on a longitudinal study of consecutive patients, who underwent trabeculectomy for surgical lowering of IOP at the Vilnius University Hospital Santaros Klinikos (2014–2017) in Lithuania. Details on the study design and the procedures can be found in previous publications^36,37^. Briefly, 130 glaucomatous eyes were enrolled to this study based on the following inclusion criteria: (1) clinical diagnosis of primary or secondary glaucoma; (2) indicated trabeculectomy because of observed progression of glaucoma or at high risk of progression due to high IOP; (3) best-corrected visual acuity of ≥ 0*.*1; and (4) refractive error from − 6*.*0 D to + 6*.*0 D of sphere and ± 3*.*0 D of cylinder. Finally, 105 glaucomatous eyes of 100 patients were used after removing the cases with pre-perimetric glaucoma, poor OCT quality, failed trabeculectomy, postoperative complications, and inadequate number of VFs/OCTs measurements or duration of follow-up.

Glaucoma was defined based on the presence of glaucomatous optic neuropathy (neuroretinal rim thinning, notching or RNFL defects) with associated glaucomatous VF defect. In addition to a clinical examination including Goldmann applanation tonometry to measure intraocular pressure (IOP), standard automated perimetry based on 30-2 Swedish Interactive Threshold Algorithm Standard strategy (Humphrey VF analyzer; Carl Zeiss Meditec, Dublin, CA, USA), and spectral-domain OCT imaging (Heidelberg Spectralis; Heidelberg Engineering, Dossenheim, Germany) were performed.

The IOP measurements, OCT imaging and VF testing were performed at five visits—baseline, and postoperatively at 3 months (M3), 6 months (M6), 9 months (M9) and 12 months (M12). The OCT imaging involved 15 × 10° rectangle scan centered on the optic nerve head with each OCT volume consisting of 49 serial horizontal B-scans scans (4.5 mm long lines, 40 images averaged) spaced at approximately 63 µm intervals. The RNFL thickness was measured from a circumferential OCT scan of 3.4 mm diameter centred at the ONH as provided by the software of the manufacturer. The VF tests were considered reliable if false positive and false negative errors were < 33% and fixation losses < 20%. The baseline test was conducted twice to prevent potential learning effects and the second VF report was used for further analysis. In this work, we used VF measurement of VF mean deviation (MD), where MD reflects the overall depression of the VF and is calculated as a weighted average decibel deviation from age normal database. As MD value goes lower, visual function of the patient becomes more damaged.

A decline in VF MD of more than 3 dB was used as glaucoma progression criterion. Here, 3 dB threshold was chosen to identify fast progressing glaucoma patients as per advice from clinical experts and also to address the class imbalance issue, which arises if the number of samples belonging to different classes (Class-1: VF decline < 3 dB, Class-2: VF decline > 3 dB) is highly skewed. A higher threshold greater than 3 dB can identify faster progressing cases, but the number of such cases is small (minority class), making it a class imbalance problem for the deep learning model. This results in models that have poor predictive performance, specifically for the minority class. Hence, a threshold of 3 dB was chosen to alleviate the class imbalance problem while identifying the fast progressing glaucoma cases.

### Proposed deep learning framework

The overview of the proposed deep learning model for glaucoma progression prediction is shown in Fig. 1. Firstly, a generative model (GAN) is utilized to synthesize OCT images at a future time-point (M6, M9, M12) conditioned on the baseline images. Next, a multimodal deep learning-based model is used to predict ∆VF class (slow vs fast progressor) at M12 based on baseline, M3, M6 and M9 data, which comprises OCT images, IOP, and VF MD values, combined with synthetic images. We performed a set of experiments to evaluate the model performance under different scenarios: (1) to compare the relevance of different input modalities in predicting glaucoma progress, (2) to ascertain the effect of adding data from more patient visits and test how early can glaucoma progress be predicted accurately, and (3) to test the effect of training the model on future synthetic OCT images along with past inputs on progression prediction performance. The statistical significance of results obtained using these different schemes of model training was determined by performing *t*-test and computing the P values, where we considered a P value of less than 0.05 as statistically significant.

### Multimodal deep learning model

To account for different input modalities and their effects on glaucoma prediction, we used a multimodal model to learn ∆VF progression. We used an approach of extracting modality specific features first and then performing late fusion of different sources of temporal data as well as patient’s baseline data to learn the common distinctive features for the classification task. The three time series modalities (OCT, IOP, ∆VF) at regular time steps (i.e., baseline, M3, M6 and M9) were fed separately into the model, along with baseline patient characteristics. Firstly, a CNN was used to learn the local features from OCT B-scan images, followed by an LSTM model to learn the temporal relationships between the OCT image features at different time-points. Secondly, ∆VF and IOP values recorded at each patient visit were fed to separate LSTM models to learn the temporal features within a single time series. The demographical and clinical characteristics of each patient taken on the first visit (baseline) were processed by a fully connected (FC) layer with ReLU activation function to extract representative deep features. Finally, the deep features learned from all networks processing different modality inputs were fused and fed to an FC layer with ReLU activation to extract the common features. This was followed by an FC layer and finally sigmoid activation function to generate the classification output to predict if a glaucoma patient is slow or fast progressor. Next, we give the details of the deep learning models comprising the progression prediction pipeline.

### CNN for extracting image features

A CNN was used to learn the local spatial features from OCT images. To alleviate the problem of limited patient data, we utilised transfer learning by using ResNet architecture as the backbone of the CNN model. ResNet network was initialized with the weights based on pre-training on ImageNet dataset. This model has been shown to perform well in medical image classification problems^38^ and hence, was chosen for our OCT image-based prediction model. The ResNet network uses convolutional and max-pooling layers to create a deep network that can learn the intricacies of a given image. At the end of the convolutional layers, the data is flattened, and an FC layer is applied to convert the convolution features to feature vectors. Therefore, a pre-trained CNN model was used to extract feature vectors for each B-scan of the OCT volume data for each patient visit, resulting in multi-dimensional time-series data corresponding to patient OCT images.

### RNN for learning temporal relationships

An RNN was used to capture the temporal dependencies in the sequential OCT, VF and IOP data collected over recurring patient visits. The RNNs learn from sequential data by utilising hidden state acting as the memory of the network by combining information from prior inputs to influence the current input and output. In this work, we have used a special type of RNN, an LSTM network, which is capable of learning long-term dependencies^39^ by utilizing “cells” in the hidden layers to regulate the flow of information These cells have three gates—an input gate, an output gate, and a forget gate. These gates determine which information needs to be retained to predict the output of the network.

### GAN for synthesis of future images

We have used a pix2pix conditional GAN^40^ for translating OCT images in time by synthesizing OCT image at follow-up visit conditioning on the OCT image from earlier patient visits. The generator network of the pix2pix GAN is based on U-Net architecture which uses an encoder-decoder type structure, along with skip connections. The encoder forms the contraction path, which allows to capture the context in the image by using convolutional and max pooling layers. The second expanding path is the decoder, which uses transposed convolutions to enable precise localization.

The discriminator of pix2pix GAN uses patch-wise method that only penalizes structure at the scale of patches. While most complex discriminators in GAN architectures utilize the whole image for establishing a synthetic or real (0 or 1) value, the patch GAN tries to classify if each N × N patch in an image is real or synthetic. The N × N patch size can vary for each specific task, but the ultimate final output is the average of all the responses of the patches considered. The primary advantages of the Patch GAN discriminator occur from the facts that they have fewer training parameters, run faster, and can be applied to arbitrarily large images.

## Data Availability

The dataset used for the analysis in the current study is not publicly available due to the terms of consent to which the participants agreed but is available from the corresponding author on reasonable request.
